# Co-Operation Invited

**Published:** 1911-10-14

**Authors:** 


### Co-operation Invited.

### WHO WILL LEND A HAND?

The supporters of this paper have shown such a generous interest in last week's developments and the reduction of the price to one penny, that we are confident they will welcome the announcement that the changes have been received with satisfaction by institutional workers everywhere throughout the country. We have had useful suggestions as to future developments, and shall welcome communications from everyone who has an idea to formulate or a request to make, whereby The Hospital can be made more perfect and more generally useful.

We propose to encourage workers to look to The Hospital whenever they have a difficulty, either in regard to a patient or to their own special work or interests.

We are anxious, too, to encourage the literary gifts and powers of observation which work in a great institution excites in many people, by inviting contributions or letters embodying matters of interest, details of practical importance and developments, with whenever possible something to make the world better by exciting a little wholesome laughter.

Numbers of workers are now adept photographers, and to these we would say send us a snapshot in duplicate of any special appliance, or article of furniture, or fittings, which may form a feature of your hospital or institution. We should also welcome photographs of the gardens, façades, and any attractive view of some portion of the hospital buildings. Each photograph may be accompanied by a short description, and everything so sent which may find a place in future numbers of The Hospital will be paid for.

We want to make our columns of practical interest and value to every worker, and the most effective way to secure this end is to enlist the co-operation of institutional workers in some, or all, of the ways here suggested.

